# Office Notes

**Published:** 1870-12

**Authors:** W. H. Shadoan


### OFFICE NOTES.

BY W. H. SHADOAN, D. D. S.

I propose during the present winter to contribute to the REGISTER short articles on cases as they occur in my practice. These articles may, and doubtless will not be very interesting to many; they are intended more for the younger members of the profession, who may be more deeply interested in them than older operators:

CASE 1. W. W. Sawyers, of Barboursville, Ky. (I will give the names of gentlemen patients by permission, so that should any of your readers wish at any time to examine the work, they having an opportunity to do so, can see for themselves just what was done), a gentleman thirty-nine years of age—good health—teeth solid and firm.

*First.* Second left inferior molar, anterior approximal and masticating surface decayed. The crown cavity was filled, but the fissures showed evident signs of decay. The first molar, which was also decayed, stood about half a line higher than the second molar.

*Method.* The intervening dentine between the masticating surface of the first molar and the cavity of decay, which was situated a line or so below the surface, was cut away freely; thus opening up the way not only to the cavity beneath, but also to the anterior cavity of the second molar, which extended down to near the gum. The gold filling was removed from the central crown cavity and the fissures freely opened; the one extending anteriorly into the approximate cavity. This done, the rough friable edges of the approximate cavity were removed to sound, firm tooth substance, when the cavity was finished the usual way. The side walls were slightly undercut, the bottom or floor of the approximate cavity was decayed a little deeper within than at the enamel surface, so that the removal of all decayed and soft tooth substance and the drilling of two small retaining pits at each angle of the bottom of the cavity was all that was necessary to complete the operation. It is well to be borne in mind that I never consider a cavity finished until the sharp edge all around the orifice of the cavity has been nicely dressed or scraped down, removing all of that portion liable to be broken or crumbled away by the plugger in the operation of filling. The rubber dam was now adjusted and the cavities well dried; first by cotton wound around an excavator, and then by wads of bibulous paper, and the bottom of the cavity wiped out by a small swab of cotton, saturated in creosote or carbolic acid, and redried with paper. The pits were filled with No. 4 Dunlevy's gold foil and then they were joined to each other by building across from the one to the other. Upon this foundation the building is made until the anterior cavity is filled up to the bottom of the opened fissure connecting the anterior with the crown cavities. Then the cavity in the masticating surface was filled, carrying the gold first into the posterior fissures and the lingual and the buccal fissures, carrying the gold across and filling the central cavity at the same time, and as the anterior portion of the filling in the crown cavity reaches the anterior fissure, it is carried forward uniting with the anterior portion of the filling, when they are all carried along together, finishing the whole at the union of the anterior cavity with the crown surface and the anterior crown fissure. The approximal cavity is broad, occupying at least one-half of the approximal wall of the tooth, extending at least a line into the crown of the tooth. The entire filling was made with No. 4 gold, only finishing off the surface with No. 60 gold of my own make. The instruments I used in filling this tooth, were of Dr. C. R. Butler's pattern: No. 2 for filling the pits, following by Nos. 3 and 5; by these the gold can be solidly impacted into every portion of the cavity as well as down firmly against the cervical wall; for this purpose No. 5 or 8 are admirably adapted. The building is made with No. 2 next the walls, and the body with No. 6, 7, or 9, though I rarely use No. 9 for this class of building. For finishing the borders of the filling, which should always be done as the filling progresses unless there is a greater amount of room than I had in this case, Nos. 5 and 7, or one of Dr. Varney's pluggers very much like No. 5, except there is no heel to the Varney plugger. For filling the crown and fissures Nos. 12 and 2, and one foot instrument for the fissures some thinner than those usually seen in the sets now offered for sale, are all that are needed, No. 10 foot for leveling down the approximal surface, and a flat foot burnisher complete the filling. Dress off the surface with files, burrs, and stone down, leaving a polished rather than a burnished surface. For this last I use pulverized pumice stone.

